# Feasibility of 3D black-blood variable refocusing angle fast spin echo cardiovascular magnetic resonance for visualization of the whole heart and great vessels in congenital heart disease

**DOI:** 10.1186/s12968-018-0508-1

**Published:** 2018-11-26

**Authors:** Markus Henningsson, Riad Abou Zahr, Adrian Dyer, Gerald F. Greil, Barbara Burkhardt, Animesh Tandon, Tarique Hussain

**Affiliations:** 1 School of Biomedical Engineering and Imaging Sciences, King’s College London, London, UK; 2 Departments of Pediatrics and Radiology, University of Texas Southwestern/Children’s Health, Dallas, TX USA

**Keywords:** Black-blood CMR, Volumetric CMR, Fast spin-echo, Whole-heart imaging

## Abstract

**Background:**

Volumetric black-blood cardiovascular magnetic resonance (CMR) has been hampered by long scan times and flow sensitivity. The purpose of this study was to assess the feasibility of black-blood, electrocardiogram (ECG)-triggered and respiratory-navigated 3D fast spin echo (3D FSE) for the visualization of the whole heart and great vessels.

**Methods:**

The implemented 3D FSE technique used slice-selective excitation and non-selective refocusing pulses with variable flip angles to achieve constant echo signal for tissue with T1 (880 ms) and T2 (40 ms) similar to the vessel wall. Ten healthy subjects and 21 patients with congenital heart disease (CHD) underwent 3D FSE and conventional 3D balanced steady-state free precession (bSSFP). The sequences were compared in terms of ability to perform segmental assessment, local signal-to-noise ratio (SNR_l_) and local contrast-to-noise ratio (CNR_l_).

**Results:**

In both healthy subjects and patients with CHD, 3D FSE showed superior pulmonary vein but inferior coronary artery origin visualisation compared to 3D bSFFP. However, in patients with CHD the combination of 3D bSSFP and 3D FSE whole-heart imaging improves the success rate of cardiac morphological diagnosis to 100% compared to either technique in isolation (3D FSE, 23.8% success rate, 3D bSSFP, 5% success rate). In the healthy subjects SNR_l_ for 3D bSSFP was greater than for 3D FSE (30.1 ± 7.3 vs 20.9 ± 5.3; *P* = 0.002) whereas the CNR_l_ was comparable (17.3 ± 5.6 vs 17.4 ± 4.9; *P* = 0.91) between the two scans.

**Conclusions:**

The feasibility of 3D FSE for whole-heart black-blood CMR imaging has been demonstrated. Due to their high success rate for segmental assessment, the combination of 3D bSSFP and 3D FSE may be an attractive alternative to gadolinium contrast enhanced morphological CMR in patients with CHD.

**Electronic supplementary material:**

The online version of this article (10.1186/s12968-018-0508-1) contains supplementary material, which is available to authorized users.

## Background

Three-dimensional (3D) cardiovascular magnetic resonance (CMR) can be used for visualization of the whole heart and great vessel morphology in patients with congenital heart disease (CHD) [1]. This is commonly achieved using cardiac and respiratory gated whole-heart 3D balanced steady-state free precession (3D bSSFP) which provides bright-blood contrast [2, 3]. However, 3D bSSFP is susceptible to signal loss due to flow-related dephasing, particularly during turbulent or rapid blood-flow [4]. Contrast-enhanced cardiovascular magnetic resonance angiography (CE CMRA) may be used to obtain volumetric, time-resolved morphological information in a much shorter scan time than 3D bSSFP. However, CE CMRA typically has lower spatial resolution than 3D bSSFP and is not synchronized with the cardiac cycle leading to motion-induced blurring. Furthermore, CE CMRA typically requires the administration of gadolinium-based contrast agents which has known (nephrogenic systemic fibrosis) and undetermined risks (related to potential retention). An alternative approach to visualize morphology in CHD is black-blood CMR, which allows for visualization of myocardium and vessel wall with positive contrast without using exogenous contrast agents by suppressing blood signal [5]. However, the conventional approach – double inversion recovery (DIR) – is limited to two-dimensional (2D) acquisitions with non-isotropic resolution [6]. Furthermore, insufficient blood-suppression with suboptimal contrast often results from slowly flowing blood or flow perpendicular to the 2D DIR slice direction.

In recent years, 3D black-blood techniques have been proposed to allow high-resolution imaging of the aortic vessel wall [7–9]. This includes fast spin echo (FSE) using variable flip angle refocusing pulses which relies on the motion sensitivity of the spin echo pulse sequence to suppress signal from flowing blood [10]. Following the 90° excitation radiofrequency (RF) pulse, a few (5–10) dummy/startup RF refocusing pulses are performed during which moving/flowing spins rapidly de-phase. The amplitude of the refocusing pulses is typically modulated during the echo-train to obtain a constant vessel wall signal [11]. To achieve short echo-spacing, non-selective refocusing pulses can be used for 3D FSE [12]. However, this may lead to artifacts arising from signal outside the field-of-view (FOV) which is excited by the non-selective refocusing pulses, and subsequently generate a free induction decay (FID) signal [13]. To avoid these artifacts, oversampling in the slice direction can be employed at the expense of increasing the scan time. Alternatively, signal averaging can be used [14], again leading to a longer total scan duration. Three-dimensional FSE of the cardiovascular system may be particularly susceptible to outer volume artifacts as it is surrounded by a significant volume of tissue from the chest and arms including subcutaneous fat. However, prolonging scan time is also undesirable due to the increased likelihood of physiological motion artifacts. Due to these technical challenges, whole-heart black-blood imaging has so far mainly employed less efficient but more motion tolerant and flow independent gradient echo acquisitions [15, 16]. A few studies have described cardiac and respiratory motion compensated black-blood 3D FSE [8, 17]. To the authors’ knowledge no systematic evaluation or optimization has been performed of important 3D FSE parameters for cardiovascular black-blood imaging.

In this study we describe a new 3D FSE protocol for black-blood CMR of the whole heart and great vessels. Although the flow sensitivity of 3D FSE is well-documented in the literature [11, 18, 19], in this study we investigate the flow suppression performance of a specific cardiovascular 3D FSE protocol in simulations and in vivo. Furthermore, we investigate if the proposed 3D FSE protocol can be performed in the absence of averaging or slice-oversampling without FID artifacts from the outer volume. The main purpose of this study was to assess the feasibility of black-blood 3D FSE for the visualization of the whole heart and great vessels in patients with CHD. We compare the new 3D FSE technique to the conventional techniques for volumetric morphological CMR – bright-blood 3D bSSFP.

## Methods

CMR studies were performed on a 1.5 T Philips scanner (Philips Healthcare, Best, The Netherlands) using a 32-channel torso coil. All participants provided written informed consent, and the study was approved by the local ethics committee (IRB STU 032016–009).

### 3D FSE pulse sequence

**Fig. 1:**
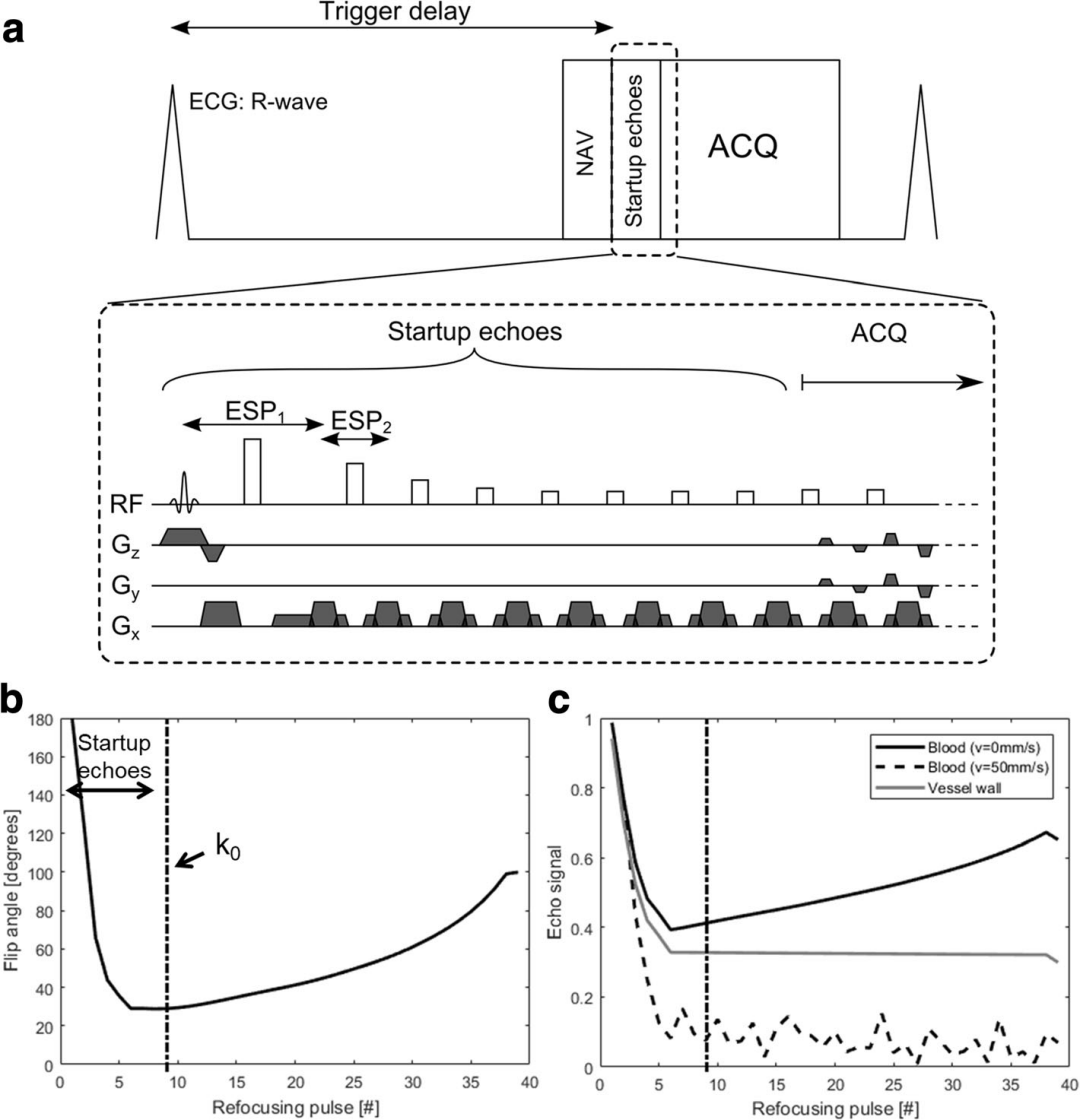
Pulse sequence diagram of 3D fast spin echo (FSE) sequence, using electrocardiogram (ECG)-triggering and respiratory navigator (NAV) for motion compensation (**a**). The slab-selective 90 excitation pulse is followed by a series of non-selective refocusing pulses, modulated to generate a constant transverse magnetisation for vessel wall tissue (T1 = 880 ms, T2 = 40 ms) (**b**). Eight startup echoes are used to discard varying signal early in the echo train, while allowing signal from flowing blood to de-phase (blood v = 0 mm/s versus v = 50 mm/s) (**c**). During the startup echoes gradients are only performed in the readout direction leading to flow-induced dephasing primarily along this direction. A centric k-space view ordering scheme is used, whereby the centre of k-space (k_0_) is acquired after the startup echoes at the 9th echo (dashed line in (**b**) and (**c**). A longer echo spacing (ESP_1_) was used between the excitation pulse and first echo compared to subsequent echoes (ESP_2_) to account for the longer duration of the slab-selective excitation pulse

The 3D FSE sequence used a slice-selective 90° excitation pulse and non-selective refocusing pulses [13]. The refocusing flip angles of the FSE were modulated to yield constant transverse magnetisation across the echo train for a specific T1 (880 ms) and T2 (40 ms) combination, assumed to be similar to those of the aortic vessel wall based on a previous study of the carotid vessel wall [20]. The first echo spacing (ESP1), from excitation pulse to the first echo, was longer than the following echo spacing (ESP2) to account for the longer duration of the slice-selective excitation pulse. ESP2 was defined as the shortest possible duration to reduce scan time and maximise vessel wall signal which has a short T2. The refocusing flip angle for one shot and the resulting transverse magnetisation for the vessel wall, static blood and flowing blood are shown in Fig. 1. A centric view-ordering scheme was used to ensure the highest temporal correlation between the respiratory navigator motion measurements and the acquisition of the centre of k-space. Eight startup echoes were used, during which no data was collected, to avoid the rapidly changing transverse magnetisation at the start of the echo train, and allow the signal from flowing blood to de-phase as shown in Fig. 1. Although the 90° excitation pulse is slab-selective, the refocusing pulses are non-selective. In practice, this means gradients are only performed in the readout-direction during the startup echoes as shown in Fig. 1a, leading to flow-induced de-phasing only along this direction. As a result, the orientation of the FOV relative to the direction of the blood flow is important to achieve black-blood contrast [21]. A coronal orientation with readout along foot-head direction was used to maximise blood signal suppression in the aorta, as determined in a small pilot study shown in Additional file 1: Figure S1.

### Simulations

To investigate the influence of flow velocity on the ability to suppress blood signal, simulations were performed using the extended phase graph algorithm. The algorithm was implemented in MatLab 2017a (MathWorks, Natick, Massachusetts, USA) with an additional phase term to simulate flow of constant velocity during the 3D FSE pulse sequence. The details of this algorithm have been described by Weigel, including the incorporation of flow [21]. Velocities from 0 mm/s to 100 mm/s were simulated to determine the flow sensitivity of the 3D FSE sequence. Acquisition parameters were identical as for those described for 3D FSE in Table 1. Relaxation times of blood at 1.5 T were used for these simulations (T1 = 1600 ms; T2 = 250 ms).

**Table 1:** Imaging Parameters for healthy subject CMR studies

	3D FSE	3D bSSFP
TR	RR-interval	4.2 ms
TE_eff_ (ms)	30.0	2.1
Echo-spacing (ms)	3.0	–
FOV (mm^3^)	300 × 300 × 110	300 × 300 × 110
Voxel size (mm^3^)	1.3 × 1.3 × 1.3	1.3 × 1.3 × 1.3
Slice oversampling	1.2	1.2
Number of startup echoes	8	10
Pixel bandwidth (Hz)	1657	1238
Number of averages	1	1
Orientation	Coronal	Coronal
SENSE acceleration	2 (PE)	2 (PE)
Respiratory gating	7 mm G + 0.6 T	7 mm G + 0.6 T
Acquisition window (ms)	140	140
Fat suppression	SPIR	SPIR
Nominal scan time	4 min 7 s	4 min 16 s

*bSSFP* balanced steady state free precession, *FH* foot-head, *FOV* field of view, *FSE* fast spin echo, *PE* phase encoding, *SENSE* sensitivity encoding, *G* gating, *T* tracking, *TE* echo time, *TR* repetition time, *SPIR* spectral presaturation with inversion recovery

### CMR studies: Outer volume artifacts

To investigate the susceptibility to outer volume artifacts of the 3D FSE sequence without signal averaging, CMR experiments were performed in a T1 phantom [22] and 3 healthy subjects (29 ± 4 years). In these experiments the slab-selective pulse excited a volume of tissue half of the size of the encoded field-of-view. The experiments aimed to spatially encode potential FID artifacts arising from tissue outside the excited FOV which only experience the non-selective refocusing pulses. Encoding a volume twice the size of the slab-selection profile is the equivalent to using a slice oversampling factor of 2. Although the slices outside the excited volume are typically discarded, in these experiments the outer slices were visually inspected for potential FID artifacts. The imaging parameters for the scans were: encoded FOV = 330 × 330 × 200 mm^3^, RF excitation profile width = 100 mm, effective echo time = 30 ms, echo-spacing = 2.7 ms, voxel size = 1.5 × 1.5 × 1.5, echo train length = 35 (plus 8 startup echoes), number of averages = 1, coronal orientation, SENSE acceleration = 2 left-right (LR). The in vivo scan were electrocardiogram (ECG)-triggered to the mid-diastolic rest period and used a diaphragmatic navigator with 7 mm gating window and 0.6 tracking factor for respiratory motion compensation.

### Healthy subject experiments: 3D FSE versus 3D bSSFP

A study was performed in 10 healthy subjects (28 ± 4 years) to compare 3D FSE and bright-blood 3D bSSFP in terms of ability to visualise cardiovascular anatomical structures. The two scans were performed in a randomized order for each subject and the imaging parameters are summarized in Table 1. The scans were ECG-triggered to coincide with the diastolic rest period, as visually determined from a time-resolved 2D cine scan. The FOV of 3D FSE and 3D bSSFP were identical, covering the whole heart and great vessels. All scans were acquired with a pencil beam navigator for respiratory motion compensation, including a 7 mm gating window and 0.6 tracking factor.

### Patient studies: 3D FSE versus 3D bSSFP

Patients with CHD referred for CMR examination at Children’s Medical Center, Dallas, Texas, USA, between September 2017 and January 2018 were considered for inclusion in this prospective study. Patients were scanned with the proposed 3D FSE scan and the findings were compared to 3D bSSFP. As these were clinically indicated CMR examinations, 3D bSSFP was often performed after any administration of gadolinium as per clinical routine in order for improved image quality. CE CMRA was not performed in all cases and so not available for comparison. Reasons for a clinical protocol of selective CE CMRA include restriction of gadolinium to cases where it is absolutely required and because, in cases where coronary anatomy is required as paramount, this information is not available on CE CMRA. The 3D FSE imaging parameters were: encoded FOV = (250–350), number of slices 120 to 180, effective echo time = 30 ms, echo-spacing = 2.7 ms, voxel size = 1.3 × 1.3 × 1.3 mm, echo train length = 18 to 35 (plus 8 startup echoes), number of averages = 1, coronal orientation, SENSE acceleration = 2 (LR). Fat suppression was performed using spectral presaturation with inversion recovery (SPIR). The 3D bSSFP imaging parameters were: encoded FOV = (250–350), number of slices 120 to 180, echo time = 2 ms, voxel size = 1.3 mm^3^ to 1.8 mm^3^, echo train length = 22 to 40 (plus 10 startup echoes), number of averages = 1, coronal orientation, SENSE acceleration = 2 (LR). SPIR was used for fat suppression. Both 3D FSE and 3D bSSFP scans were ECG-triggered to the mid-diastolic rest period or end-systolic rest period (depending on which was longer and more consistent) and used a diaphragmatic navigator with 3–7 mm gating window (dependent on patient size) and 0.6 tracking factor for respiratory motion compensation. The scan times for both 3D FSE and 3D bSSFP were recorded.

### Image analysis

For the healthy subjects and CHD patient scans using whole-heart 3D FSE and 3D bSSFP, the relative success rates of achieving a full sequential segmental diagnosis (i.e. identifying all thoracic cardiovascular morphological elements – superior vena cava (e); inferior vena cava; coronary sinus; right atrium; left atrium; right ventricle; left ventricle; aorta; main pulmonary artery; pulmonary artery branches; pulmonary veins; aorta and head & neck branches; coronary artery origins) was compared between 3D FSE and 3D bSSFP. A structure was recorded as visualized if its connections were identified and there was no more than mild image blurring affecting that structure. More specifically, using a previously validated scoring system, the image quality score was greater than or equal to 3 out of 4 [23].

Acquisition of a noise image required for global signal to noise ratio (SNR) and contrast to noise ratio (CNR) calculations with parallel imaging was not considered practical due to time constraints [24]. Furthermore SNR and CNR between bright blood (3D bSSFP) and black blood sequences (3D FSE) have different signals of interest. Nevertheless, comparisons are provided in order to provide insight and, by ensuring imaging parameters such as the patient position, field-of-view, phase encoding direction and acceleration factor were unchanged between sequences, and by selecting identical regions-of-interest (ROIs) in both sets of resulting images, a local SNR (SNR_l_) and local CNR (CNR_l_) were calculated as detailed:

3D bSSFP SNR_l_ = IB / SD (L).

3D FSE SNR_l_ = IM / SD (L).

3D bSSFP CNR_l_ = (IB – IM) / SD (L).

3D FSE CNR_l_ = (IM – IB) / SD (L).

where IB and IM refers to the mean signal-intensity in an ROI in the blood-pool (proximal ascending aorta) and myocardium (mid ventricular septum) respectively, and SD (L) refers to the standard deviation of an ROI of air in the lungs (chosen to contain a minimum of 100pixels while avoiding any visible vascular structures).

### Statistical analysis

All continuous variables are presented as mean ± standard deviation, while non-continuous variables are presented as median [25th percentile, 75th percentile]. Differences in continuous variables were statistically compared using paired t-test with a significance thresh-hold of 0.05. Non-continuous variables were compared using Wilcoxon sign-rank test, also with a 0.05 significance thresh-hold. To account for multiple comparisons, Holm-Bonferroni correction was used.

## Results

### Simulations

**Fig. 2:**
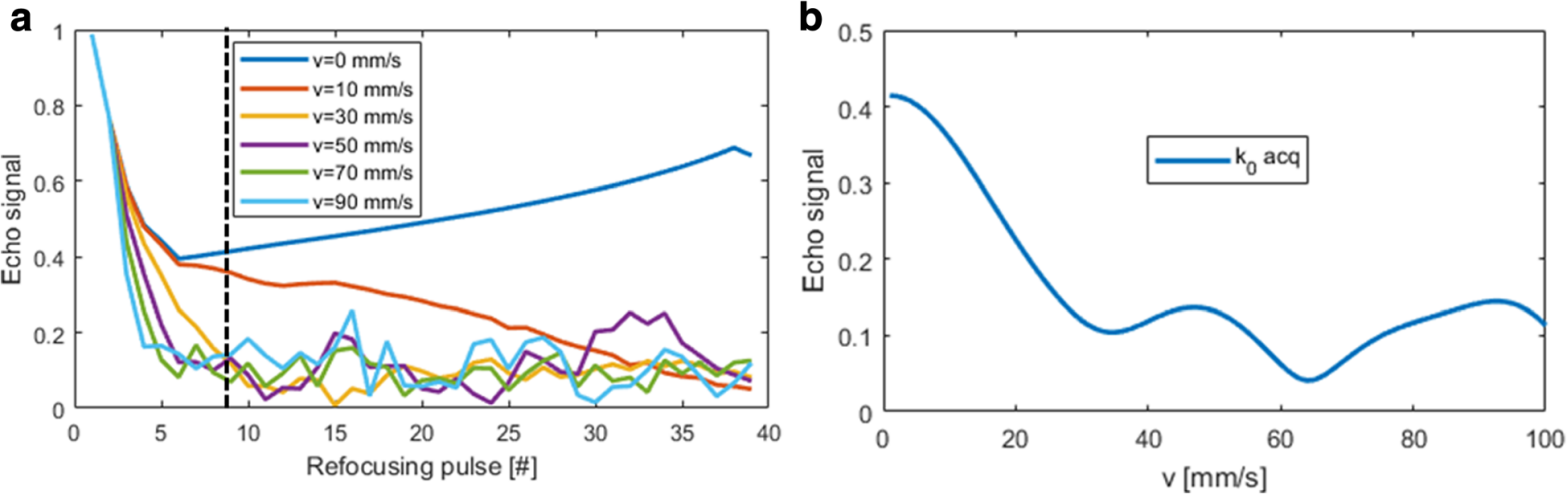
Simulated signal of blood at different velocities (in mm/s) for 3D FSE across the echo train (**a**). Eight startup echoes were used at the start of the echo train during which no data was collected, and using a centric view ordering scheme the centre of k-space (k_0_) was acquired at the 9th echo (dashed line). The echo signal at k_0_ acquisition shows good suppression of velocities higher than 30 mm/s (**b**)

Simulated transverse magnetisation across the echo train for the 3D FSE for blood with different velocities are shown in Fig. 2a. Flowing blood rapidly de-phase during the initial startup echoes leading to black-blood contrast. The echo signal at the acquisition of the centre of k-space are shown in Fig. 2b, where good blood signal suppression for velocities higher than 30 mm/s can be seen.

### Outer-volume suppression experiments

**Fig. 3:**
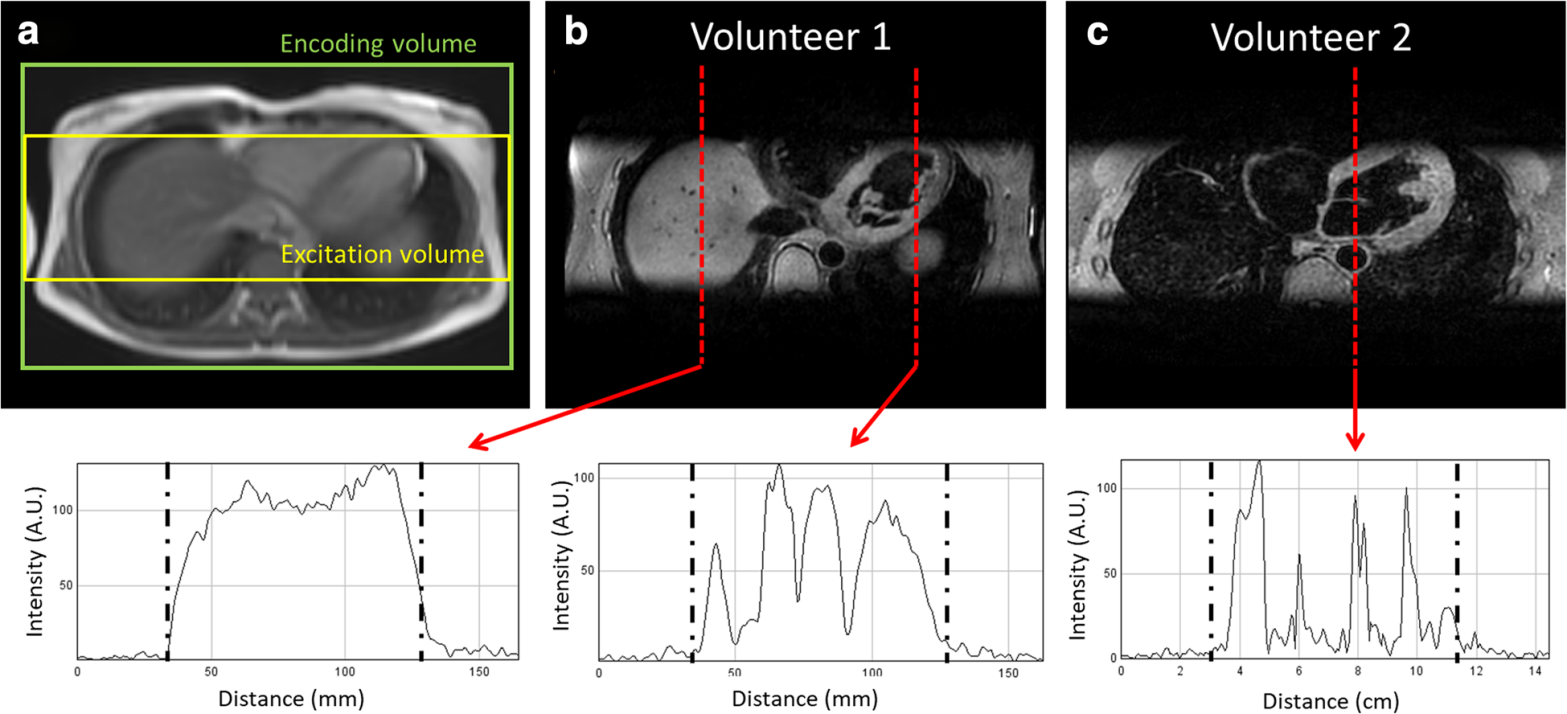
Scan planning of 3D FSE in coronal orientation, using a narrower excitation volume than encoding volume in the slice direction (**a**) to spatially encode potential artifacts arising from the non-selective refocusing pulses. Resulting 3D FSE image for two healthy subjects, demonstrating excellent suppression of outer volume tissue (**b** and **c**). Signal intensity profiles through the liver and heart highlight the difference in signal between tissue within the excited volume and outer volume which are separated in the plots by the dashed lines

The phantom experiment using twice the size of encoded FOV in slice direction compared to the excited FOV and resulting 3D FSE is shown in Additional file 1: Figure S2. Excellent suppression of signal for all vials (which ranged in T1 from 250 ms to 1500 ms) was achieved outside the volume of the 90° excitation pulse. Good outer volume suppression was also achieved in the in-vivo experiments in three healthy subjects where a similar experiment was performed with increased encoded FOV relative to the excited FOV. The 3D FSE images from two healthy subjects are shown in Fig. 3. Signal profiles in the slice excitation direction show practically no measureable signal in tissue outside the excited FOV, highlighting the excellent outer volume suppression of this technique.

### Healthy subject studies: 3D FSE versus 3D bSSFP

Three-dimensional bSSFP failed to achieve diagnostic accuracy in all cases (0% success rate). Failure was due to more than mild blurring of one or more pulmonary veins (PV’s) in 10 out of 10 cases (6 right lower, 9 right upper, 9 left upper and 4 left lower PV’s were inadequately visualized). This was invariably due to dephasing artefacts in the PVs. Two healthy subjects had poor image quality of head and neck vessels (left common carotid, *n* = 1; left subclavian artery, *n* = 1).

**Fig. 4:**
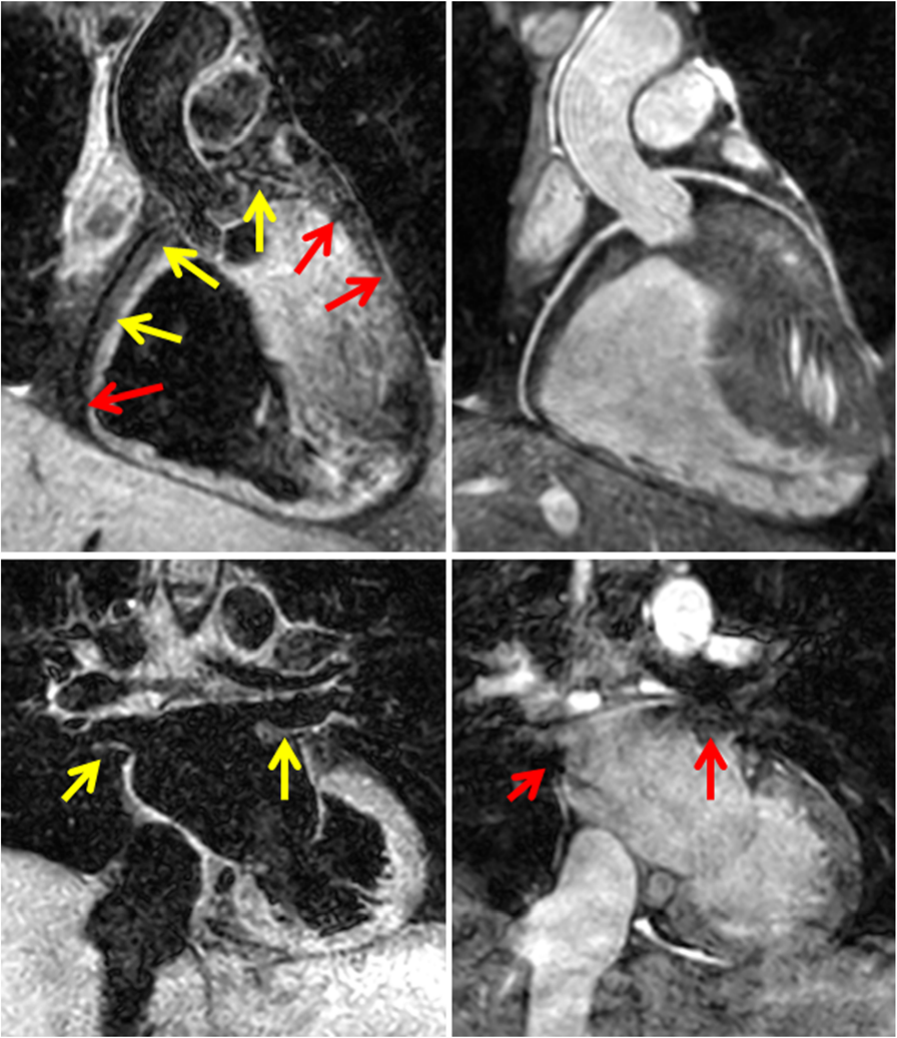
3D FSE (left) and 3D bSSFP (right) of a healthy subject, reformatted to visualise the right coronary artery and left anterior descending arteries (top images), and right and left pulmonary veins (bottom images). The proximal coronaries can be visualised with 3D FSE (yellow arrows) while distal arteries are not distinguishable. However, the pulmonary veins are clearly seen in the 3D FSE, unlike 3D bSSFP where flow artifacts hamper visualization

3D FSE achieved full segmental diagnoses in 1 case (10% success rate). All failed cases were due to inadequate quality of coronary origins while PV visualization was successful in all apart from 1 case (90% success). Additionally, the left subclavian artery appeared with more than mild blurring in 2 cases, the right subclavian artery failed in 2 cases, and the branch pulmonary arteries were inadequate in 1 case. However, the combination of 3D bSSFP and 3D FSE was able to provide the full segmental morphological diagnosis for 9 out of 10 cases (90% success). Images from one healthy subject are shown in Fig. 4, demonstrating excellent visualization of the right coronary artery (RCA) and left anterior descending coronary artery (LAD) using 3D bSSFP, while only the proximal course of the RCA and LAD are visualized with 3D FSE. However, the pulmonary veins could be visualized with 3D FSE but not 3D bSSFP. The scan time for 3D FSE was similar to 3D bSSFP (9:21 ± 2:8 vs 8:36 ± 1:54; *P* = 0.19). 3D bSSFP SNR_l_ was greater than 3D FSE SNR_l_ of the sequence (30.1 ± 7.3 vs 20.9 ± 5.3; *P* = 0.002) whereas the CNR_l_ was comparable (17.3 ± 5.6 vs 17.4 ± 4.9; *P* = 0.91).

### Patient studies: 3D FSE versus 3D bSSFP

Twenty one CHD patients underwent CMR (12 female; age 9.6 ± 7.4 years (range 9 months to 26 years)). The mean heart rate was 84 ± 15 bpm for 3D FSE and 87 ± 16 bpm for 3D bSSFP. There was no significant difference between heart rates between sequences (*P* = 0.49). The scan time for 3D FSE was similar to 3D bSSFP (3D FSE = 5:17 ± 1:33 vs 3D bSSFP = 4:48 ± 1:55; *P* = 0.46). The main indication for imaging was coronary anatomy in 5 cases, PV anatomy in 4 cases, aortic root anatomy in 1 case and complex single ventricle anatomy in 3 cases. The remaining cases received imaging for follow-up of repaired truncus arteriosus, tetralogy or transposition.

In patients with CHD, 3D bSSFP failed to achieve full segmental diagnoses in all but 1 case (5% success rate). Failure was due to more than mild blurring of one or more PV’s in 15 out of 21 cases (12 right lower, 9 right upper, 6 left upper and 3 left lower PV’s were inadequately visualized). This was invariably due to dephasing artifacts in the PVs. Three patients had poor image quality of head and neck vessels (3 right subclavian arteries, 2 right common carotid arteries, 1 left common carotid and 1 left subclavian artery were not seen adequately). Pulmonary arteries showed more than mild blurring in 3 cases (all with stenotic lesions causing dephasing artifacts). In two cases, the SVC had more than mild blurring.

**Fig. 5:**
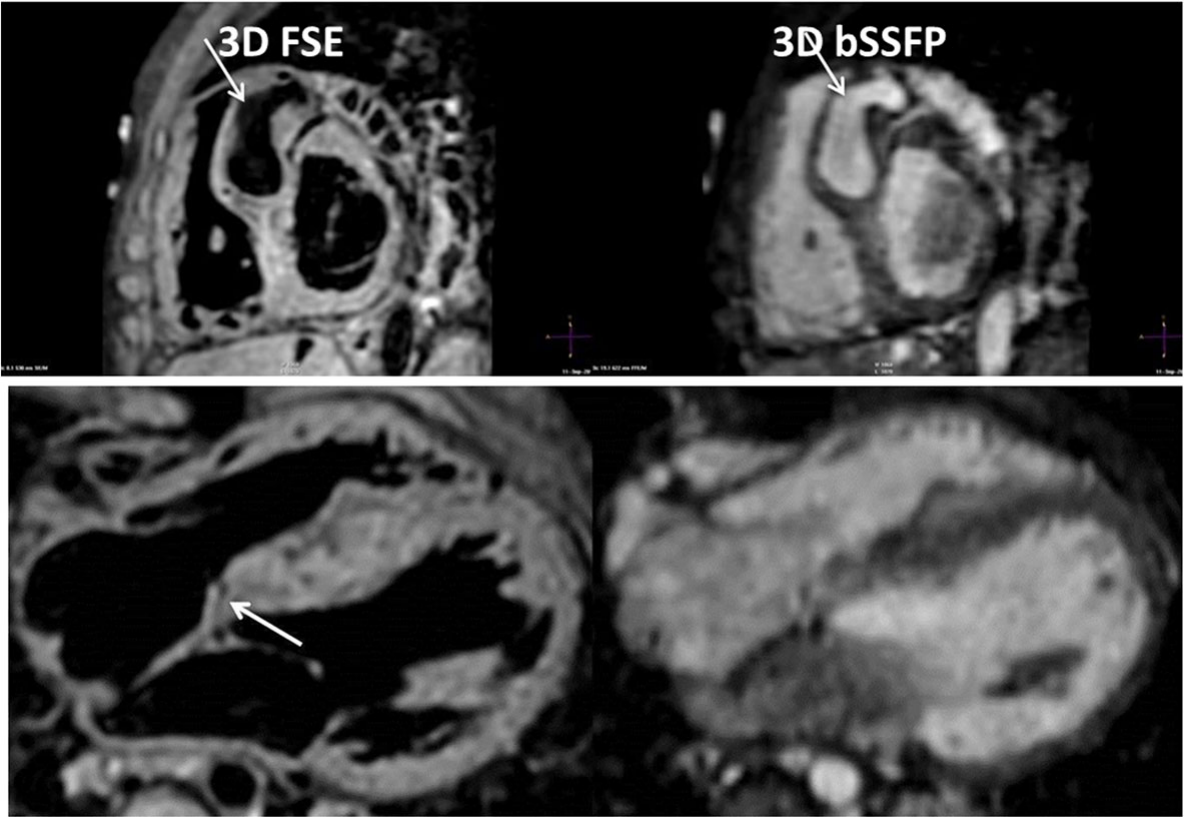
3D FSE (left) and 3D bSSFP (right) images of a 4 year old patient with a large interventricular septal coronary pouch adjacent to the left anterior descending artery (arrows, top images). The patient also had a coronary fistula from the right coronary artery which could be visualized with 3D FSE (arrow, bottom left) but not 3D bSSFP (bottom right)

**Fig. 6:**
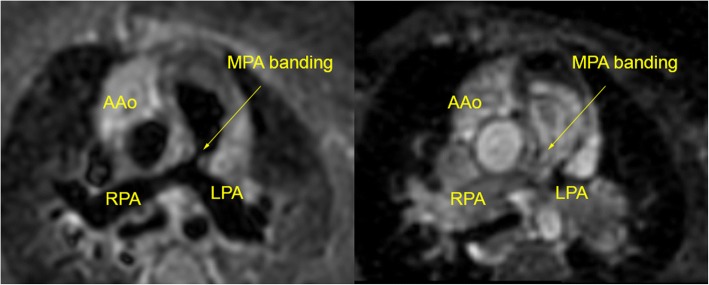
3D FSE (left) and 3D bSSFP (right) images of a 6 month old patient with a pulmonary artery band (as palliation for an unbalanced atrioventricular septal defect). The pulmonary artery anatomy (which is vital for subsequent surgical planning) is clearly seen with 3D FSE (arrow) but not 3D bSSFP (right) due to dephasing artifact from turbulent flow after the band. The patient went on to have an uneventful cavopulmonary anastomosis. AAo = ascending aorta; MPA = main pulmonary artery; RPA = right pulmonary artery; LPA = left pulmonary artery

**Fig. 7:**
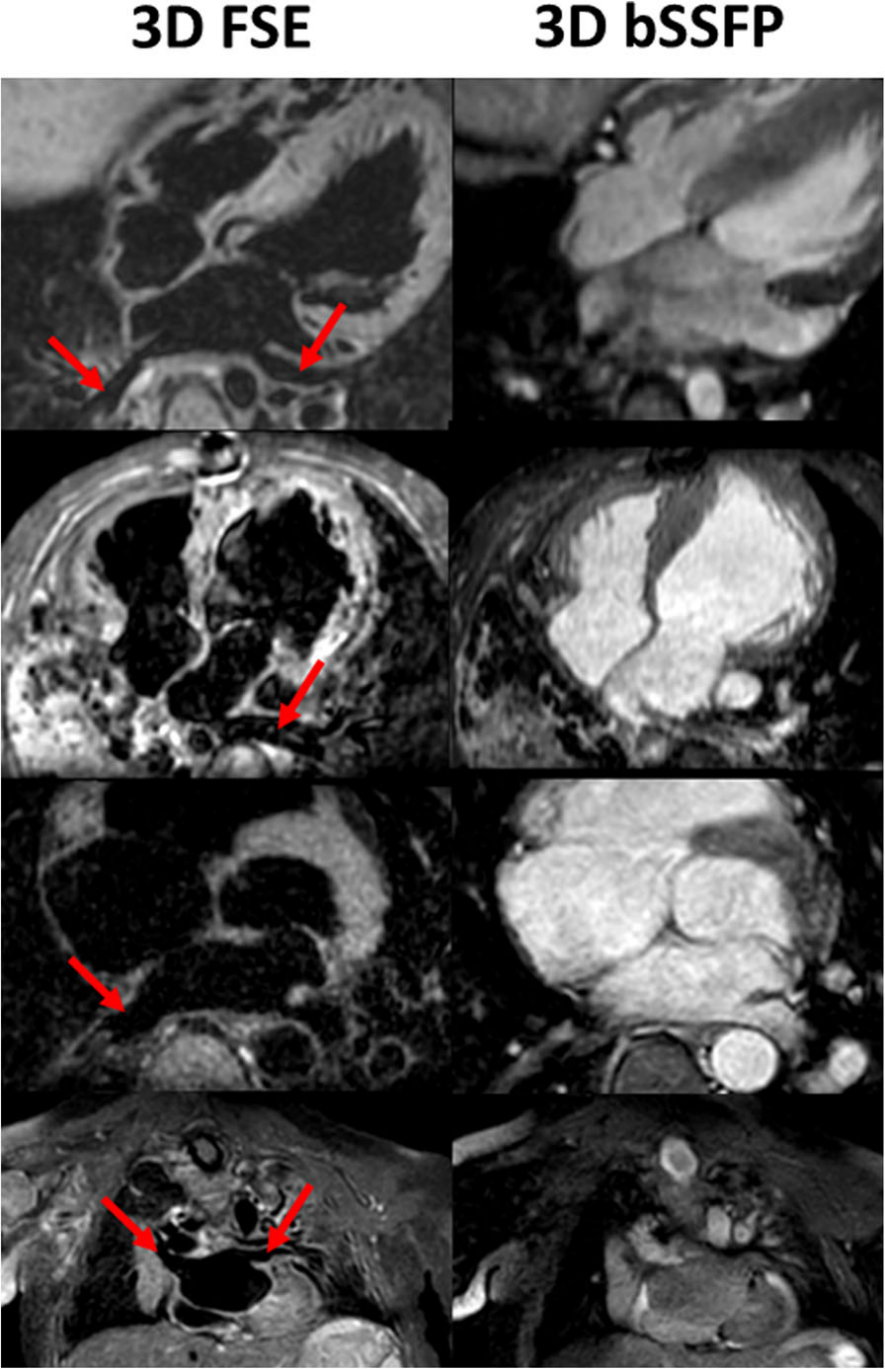
Example images from four patients with congenital heart disease. 3D FSE images are shown on the left, 3D bSSFP images on the right hand side for each patient. Red arrows mark the pulmonary veins which are well visualized on 3D FSE, but not sufficiently or not at all on 3D bSSFP

In contrast, 3D FSE achieved full segmental diagnoses in 5 cases (23.8% success rate). All failed cases were due to inadequate image quality of coronary origins while PV visualization was successful in all cases. Additionally, the coronary sinus had more than mild blurring in 2 cases and the left subclavian artery visualization failed in 2 cases. The combination of 3D bSSFP and 3D FSE was able to provide the full segmental morphological diagnosis for all cases. A patient with a coronary fistula, which could be visualized with 3D FSE but not 3D bSSFP, is shown in Fig. 5. A case where pulmonary arteries were visualized with 3D FSE but not 3D bSSFP (due to dephasing artifact from a pulmonary artery band) is shown in Fig. 6. Example images from 4 CHD patients with improved visualization of PVs are shown in Fig. 7.

Segmental anatomy, based on the 3D FSE and 3D bSSFP sequences, was considered accurate in all cases where visualization was possible. Thirteen cases had known anatomy from previous surgery and imaging, in all cases segmental diagnosis was accurate based on 3D FSE and 3D bSSFP sequences. Two cases required CMR as the PV anatomy was not known. In both cases the segmental diagnosis was accurate based on the 3D FSE and 3D bSSFP sequences as confirmed on subsequent surgery. Three cases had normal segmental anatomy (1 fistula, 1 Kawasaki disease and 1 bicuspid aortic valve) on echocardiographic imaging and this was also shown on independent analysis using the 3D FSE and 3D bSSFP sequences. These 3 cases did not have surgical confirmation. A further 3 cases required CMR for uncertain coronary origins based on echocardiography. In all these 3 cases, coronary anatomy was demonstrated by CMR based on the 3D bSSFP sequences. Two of these cases had abnormal coronary artery origins that are known to be benign variants and thus do not have surgical confirmation. The remaining case had normal coronary origins based on the 3D FSE and 3D bSSFP sequences. All 3 patients who underwent CMR to determine coronary origins and course were discharged from further follow-up based on this assessment.

Both the SNR_l_ (42.9 ± 12.0 vs 23.1 ± 9.8; *P* < 0.001) and the CNR_l_ (26.6 ± 7.6 vs 19.3 ± 8.5; *P* = 0.006) were higher for 3D SSFP vs 3D FSE.

## Discussion

In this study, we have demonstrated the feasibility of 3D FSE for black-blood imaging of the whole heart and great vessels. In particular, 3D FSE is better at visualizing the PVs compared to conventional anatomical 3D CMR using bright-blood bSSFP. However, 3D FSE is inferior compared to 3D bSSFP for coronary artery imaging.

3D FSE has been widely used for brain [25–28] or musculoskeletal imaging [29–31]. Recent pulse sequence improvements, using non-selective refocusing pulses with variable flip angles allow rapid data acquisition with improved point-spread functions. However, despite its many advantages, 3D FSE for CMR has remained limited to aortic vessel wall imaging [7, 8]. Aortic imaging is technically less challenging than whole-heart imaging because both respiratory and cardiac motion conditions are more benign in the aorta compared to cardiac structures. An important step for enabling whole-heart 3D FSE is therefore to introduce ECG-triggering and respiratory motion compensation, to minimize motion-induced de-phasing. To minimize respiratory motion, a conventional respiratory navigator approach was employed using gating and tracking. Although further improvements in image quality may be achieved using more advanced respiratory motion compensation techniques such as self-navigation [32] or image-based navigation [33, 34], in this study only pulse sequence features which are available on all major vendors’ scanners were employed.

In the phantom study, we demonstrate that good flow suppression can be achieved for blood flow aligned with the readout gradient for velocities of 30 mm/s. This velocity spoils approximately 75% of the blood signal, as shown in Fig. 2b. Comparable flow sensitivity is achieved with 2D DIR, which relies on flow perpendicular to the slice direction, assuming 16 mm slice re-inversion width and 400 inversion time. For 3D FSE the de-phasing of the blood signal occurs during the startup echoes which take approximately 30 ms, and in the simulations we have only considered constant velocity flow during this time. Higher order motion will yield further de-phasing. Improved flow suppression performance may be achieved by using additional startup echoes, extending the startup period, at the expense of reducing time during the cardiac rest period available for image acquisition.

Due to the need for close temporal proximity between respiratory motion estimation and acquisition of centre of k-space, a centric profile order is almost exclusively used for navigator gated, high-resolution CMR [35]. A centric profile order could potentially cause FID artifacts from tissue outside 90° excitation volume that only experiences the refocussing pulses which, apart from the first refocussing pulse, are all smaller than 180°. To mitigate FID artifacts 3D FSE protocol employ signal averaging, or conservative slice oversampling (increased number of slice encoding steps) which leads to increased scan time [13]. However, as demonstrated in the phantom and in vivo experiment with slice excitation width half of the encoded ROV, practically no signal was observed in tissue experiencing only non-selective refocusing pulses. This justifies the use of a low slice oversampling factor (1.2 was used to account for imperfections in slice excitation profile) and the absence of signal averaging. As a result, the scan time of the 3D FSE was comparable to the 3D bSSFP scan in both healthy subjects and patients. Additional scan time reduction could be achieved using outer volume suppression [36, 37]. With this approach further suppression of peripheral tissue may be achieved by incorporating a slab-selective RF pulse for the first refocusing pulse, orthogonal to the excitation pulse, resulting in only a 2D selective “inner volume” experiencing both excitation and refocusing [12, 38].

As demonstrated in the patient study, 3D FSE offers very clear advantages in pulmonary vein imaging compared to 3D bSSFP, while 3D bSSFP provides superior coronary artery imaging. The combination of 3D bSSFP and 3D FSE was very successful in achieving a full sequential segmental diagnosis in CHD. In particular, structures that are prone to dephasing artifact in 3D bSSFP, such as stenotic lesions and pulmonary veins, tended to show good blood suppression and superior image quality in the 3D FSE images. This made the acquisition of both sequences complementary in achieving a full segmental diagnosis. Other studies reviewing the rate of full segmental diagnosis of 3D bSSFP show a higher rate of success [39, 40], but these studies define success merely as operator confidence in segmental connections.

Our study defined success as only mild blurring or no blurring but showed high success rate overall using the combination of non-contrast 3D ECG-triggered sequences. However, proximal coronary artery visualization in the 3D FSE images was poor in most cases. This is likely due to the orientation of the coronary arteries relative to the readout direction. Due to the complex geometry of the coronary arteries, and the importance of aligning the readout direction with the direction of flow to achieve blood suppression, there is unlikely to be an orientation which allows good depiction of all three arteries in a single whole-heart acquisition. Gradients may be inserted during the startup echoes along the phase- and slice-encoding directions to achieve flow suppression in all three dimensions. However, the gradients should be balanced within each echo-spacing to ensure constructively aligned spin echoes and stimulated echoes. As the readout gradient strength is defined as the maximum possible to minimize echo-spacing and acquisition time, the use of additional gradients may result in lowered achievable readout gradient strength and longer echo-spacing, and susceptibility to eddy currents. Alternatively, magnetisation preparation pulses such as motion sensitized driven-equilibrium [41, 42] or delay alternating with nutation for tailored excitation [43] could be used to de-phase flowing blood in any direction. Motion sensitized driven-equilibrium achieves black-blood contrast by adding strong de-phasing gradients within a T2-preparation module. However, due to the short T2 of the myocardium and vessel wall this will introduce a significant SNR penalty. Delay alternating with nutation for tailored excitation achieves blood suppression by performing a series of phase-altering, small flip angle RF pulses with gradients in between, exploiting the large steady state signal difference between static and moving spin. Although this results in less T2-weighting, a large number of RF pulses are required to reach steady-state. As a result, the pre-pulse is relatively long (approximately 100–200 ms) which reduces the time available for imaging within the cardiac cycle. In general, any flow dependent pre-pulses have to be performed during the cardiac rest period to avoid motion-induced signal loss which is a limitation of such approaches. An alternative to using pre-pulses may be to use a targeted oblique thin-slab 3D FSE acquisition for each coronary artery [44], and ensure that readout-direction is aligned with the main course of the coronary artery to ensure maximum blood suppression. Improved coronary conspicuity may also be achieved by disabling the fat suppression pulse which could yield high contrast between the epicardial fat embedding the coronary arteries and the suppressed signal from the coronary lumen. Recently, an intrasvascular iron-based contrast agent, ferumoxytol, has been demonstrated to improve blood suppression for 2D black-blood FSE [45]. A similar approach may prove beneficial to improve blood suppression for 3D FSE as well, due to the significantly shortened T2 of the blood pool.

Previous studies using 3D bSSFP have demonstrated excellent image quality for pulmonary vein imaging [46, 47], which differs significantly from the findings of this study. Both previous studies include patients with atrial fibrillation which typically have lower flow through the pulmonary veins, leading to more favourable conditions for 3D bSSFP compared to our paediatric and healthy subject cohort. Secondly, both previous studies use non-standard 3D bSSFP protocol with non-selective RF pulses, which has the favourable property of allowing for shorter echo time and repetition time. This in turn leads to less de-phasing between RF pulse and echo, as well as less de-phasing between RF pulses, and subsequently higher signal in the presence of flow compared to conventional slab-selective RF-pulses. The drawback of non-selective pulses is that the field-of-view must be large enough to encompass the entire torso to avoid wrap from excited chest wall and back signal. This can lead to an increased scan time which is undesirable. In this study we have used standard 3D bSSFP protocol with slab selective RF pulses, which appears to yield flow artifacts in the pulmonary veins in this patient cohort and the healthy volunteers.

Although 3D FSE may not be used as a direct replacement for 3D bSSFP due to the unreliability for coronary artery visualisation, the combination of 3D bSSFP and 3D FSE can be used to achieve a full segmental diagnosis, and subsequently may avoid the use of contrast-enhanced CMRA. This may justify the additional scan time incurred by including 3D FSE alongside 3D bSSFP in the CMR protocol. With the advent of increased vigilance regarding the use of gadolinium-based contrast agents in children, clinicians may reduce the need for contrast agents by performing both 3D FSE and 3D bSSFP. However, further studies are required to compare the diagnostic performance of 3D bSSFP and 3D FSE compared to CE CMRA.

In the healthy subject study without gadolinium administration, the SNR_l_ of 3D bSSFP which aims to yield high signal from blood, was higher than the SNR of 3D FSE where a constant echo signal across the echo train for myocardiium (or vessel wall) is sought. However, the CNR_l_ between blood and myocardium was similar between 3D bSSFP and FSE, which indicates that the methods can yield similar differentiation of blood and myocardium, provided that slowly flowing blood signal is adequately suppressed for 3D FSE or that rapidly flowing blood signal is not de-phased for 3D bSSFP. Although the patient data shows improved SNR_l_ and CNR_l_ for the 3D bSSFP sequence, this is likely due to the administration of gadolinium in these clinically indicated examinations. However, the proposed 3D FSE may suffer from lower SNR in patients with high heart-rates as this would lead to more frequently applied excitation pulses. Triggering on alternate heart-beats may improve SNR in these cases at the expense of prolonging scan time.

A limitation of this study is that no comparison with alternative whole-heart 3D black-blood techniques was performed such as T2 prepared phase sensitive inversion recovery [48] or interleaved T2prep acquisition [15]. This was primarily due to time constraints as the gradient echo based black blood scans have a significantly longer scan time as previously described. Further studies are required to compare the merits of 3D FSE to other techniques for volumetric black-blood CMR, such as interleaved T2prep [15], T2 prepared inversion recovery [48, 49], or motion sensitized driven equilibrium [17]. The ability of 3D FSE to suppress flowing blood is limited to primarily the readout direction. This makes the orientation of the FOV relative to the blood flow an important consideration for 3D FSE. Although this was observed in the preliminary pilot study, a limitation of this study was that no systematic evaluation was performed of the ideal orientation to visualise a particular cardiac anatomy.

## Conclusion

The feasibility of 3D FSE for whole-heart black-blood CMR imaging has been demonstrated. The proposed approach enables high-resolution black-blood whole-heart CMR imaging in a scan time similar to that of the comparable bright-blood technique 3D bSSFP. In patients with CHD, 3D FSE was found to be particularly effective at visualizing the PVs but failed to visualize the coronary arteries in many cases. However, the combination of 3D bSSFP and 3D FSE whole-heart imaging improves the success rate of cardiac morphological diagnosis compared to either technique in isolation may be an attractive alternative to gadolinium contrast enhanced morphological CMR.

## Additional file

Additional file 1: **Figure S1.** The orientation of the field-of-view determines effectiveness of blood suppression as gradients are only performed in readout direction during startup echoes. As a result, for coronal orientation with readout in foot-head direction, blood signal is well-supressed in the descending aorta while residual signal is present in the aortic arch (arrows). Similarly, for a transverse orientation with readout along the anterior-posterior direction, the blood signal in the aortic arch is better suppressed while significant blood signal can be seen in the descending aorta (arrows). **Figure S2.** Scan planning of 3D FSE, using a narrower excitation volume than encoding volume in the slice direction (a) to spatially encode potential artifacts arising from the non-selective refocusing pulses. Resulting 3D FSE image shown in slice-encoding direction demonstrating excellent suppression of outer volume signal (b). In-plane images through the inner volume (c) and outer volume (d) shows the effectiveness of outer volume suppression. The signal in the outer volume image is magnified by a factor of 50 to highlight the signal suppression in these vials which ranged in T1 from 250 ms to 1500 ms. (DOCX 495 kb)

## Abbreviations

2D: Two-dimensional
3D: Three-dimensional
bSSFP: Balances steady-state free precession
CE: Contrast enhanced
CHD: Congenital heart disease
CMR: Cardiovascular magnetic resonance
CMRA: Cardiovascular magnetic resonance angiography
CNR_l_: Local contrast-to-noise ratio
DIR: Double inversion recovery
ECG: Electrocardiogram
ESP: Echo spacing
FID: Free induction decay
FOV: Field-of-view
FSE: Fast spin echo
LAD: Left anterior descending coronary artery
PV: Pulmonary vein
RCA: Right coronary artery
RF: Radiofrequency
ROI: Region of interest
SENSE: Sensitivity encoding
SNR_l_: Local signal-to-noise ratio

## References

[1] Fratz S, Chung T, Greil GF, Samyn MM, Taylor AM, Valsangiacomo Buechel ER, Yoo SJ, Powell AJ (2013). Guidelines and protocols for cardiovascular magnetic resonance in children and adults with congenital heart disease: SCMR expert consensus group on congenital heart disease. J Cardiovasc Magn Reson.

[2] Weber OM, Martin AJ, Higgins CB (2003). Whole-heart steady-state free precession coronary artery magnetic resonance angiography. Magn Reson Med.

[3] Beerbaum P, Sarikouch S, Laser KT, Greil G, Burchert W, Korperich H (2009). Coronary anomalies assessed by whole-heart isotropic 3D magnetic resonance imaging for cardiac morphology in congenital heart disease. J Magn Reson Imaging.

[4] Bieri O, Scheffler K (2005). Flow compensation in balanced SSFP sequences. Magn Reson Med.

[5] Vyas HV, Greenberg SB, Krishnamurthy R (2012). MR imaging and CT evaluation of congenital pulmonary vein abnormalities in neonates and infants. Radiographics.

[6] Edelman RR, Chien D, Kim D (1991). Fast selective black blood MR imaging. Radiology.

[7] Eikendal AL, Blomberg BA, Haaring C, Saam T, van der Geest RJ, Visser F, Bots ML, den Ruijter HM, Hoefer IE, Leiner T (2016). 3D black blood VISTA vessel wall cardiovascular magnetic resonance of the thoracic aorta wall in young, healthy adults: reproducibility and implications for efficacy trial sample sizes: a cross-sectional study. J Cardiovasc Magn Reson.

[8] Mihai G, Varghese J, Lu B, Zhu H, Simonetti OP, Rajagopalan S (2015). Reproducibility of thoracic and abdominal aortic wall measurements with three-dimensional, variable flip angle (SPACE) MRI. J Magn Reson Imaging.

[9] Wehrum T, Dragonu I, Strecker C, Schuchardt F, Hennemuth A, Drexl J, Reinhard T, Bohringer D, Vach W, Hennig J (2017). Aortic atheroma as a source of stroke - assessment of embolization risk using 3D CMR in stroke patients and controls. J Cardiovasc Magn Reson.

[10] Busse RF, Hariharan H, Vu A, Brittain JH (2006). Fast spin echo sequences with very long echo trains: design of variable refocusing flip angle schedules and generation of clinical T2 contrast. Magn Reson Med.

[11] Busse RF, Brau AC, Vu A, Michelich CR, Bayram E, Kijowski R, Reeder SB, Rowley HA (2008). Effects of refocusing flip angle modulation and view ordering in 3D fast spin echo. Magn Reson Med.

[12] Mitsouras D, Mulkern RV, Owens CD, Conte MS, Ersoy H, Luu TM, Whitmore AG, Creager MA, Rybicki FJ (2008). High-resolution peripheral vein bypass graft wall studies using high sampling efficiency inner volume 3D FSE. Magn Reson Med.

[13] Mugler JP (2014). Optimized three-dimensional fast-spin-echo MRI. J Magn Reson Imaging.

[14] Magland JF, Rajapakse CS, Wright AC, Acciavatti R, Wehrli FW (2010). 3D fast spin echo with out-of-slab cancellation: a technique for high-resolution structural imaging of trabecular bone at 7 tesla. Magn Reson Med.

[15] Andia ME, Henningsson M, Hussain T, Phinikaridou A, Protti A, Greil G, Botnar RM (2013). Flow-independent 3D whole-heart vessel wall imaging using an interleaved T2-preparation acquisition. Magn Reson Med.

[16] Varela M, Morgan R, Theron A, Dillon-Murphy D, Chubb H, Whitaker J, Henningsson M, Aljabar P, Schaeffter T, Kolbitsch C (2017). Novel MRI technique enables non-invasive measurement of Atrial Wall thickness. IEEE Trans Med Imaging.

[17] Srinivasan S, Hu P, Kissinger KV, Goddu B, Goepfert L, Schmidt EJ, Kozerke S, Nezafat R (2012). Free-breathing 3D whole-heart black-blood imaging with motion sensitized driven equilibrium. J Magn Reson Imaging.

[18] Hinks RS, Constable RT (1994). Gradient moment nulling in fast spin echo. Magn Reson Med.

[19] Storey P, Atanasova IP, Lim RP, Xu J, Kim D, Chen Q, Lee VS (2010). Tailoring the flow sensitivity of fast spin-echo sequences for noncontrast peripheral MR angiography. Magn Reson Med.

[20] Coolen BF, Poot DH, Liem MI, Smits LP, Gao S, Kotek G, Klein S, Nederveen AJ (2016). Three-dimensional quantitative T1 and T2 mapping of the carotid artery: sequence design and in vivo feasibility. Magn Reson Med.

[21] Weigel M (2015). Extended phase graphs: dephasing, RF pulses, and echoes - pure and simple. J Magn Reson Imaging.

[22] Captur G, Gatehouse P, Keenan KE, Heslinga FG, Bruehl R, Prothmann M, Graves MJ, Eames RJ, Torlasco C, Benedetti G (2016). A medical device-grade T1 and ECV phantom for global T1 mapping quality assurance-the T1 mapping and ECV standardization in cardiovascular magnetic resonance (T1MES) program. J Cardiovasc Magn Reson.

[23] McConnell MV, Khasgiwala VC, Savord BJ, Chen MH, Chuang ML, Edelman RR, Manning WJ (1997). Comparison of respiratory suppression methods and navigator locations for MR coronary angiography. AJR Am J Roentgenol.

[24] Yu J, Agarwal H, Stuber M, Schar M (2011). Practical signal-to-noise ratio quantification for sensitivity encoding: application to coronary MR angiography. J Magn Reson Imaging.

[25] Mugler JP, Bao S, Mulkern RV, Guttmann CR, Robertson RL, Jolesz FA, Brookeman JR (2000). Optimized single-slab three-dimensional spin-echo MR imaging of the brain. Radiology.

[26] Pouwels PJ, Kuijer JP, Mugler JP, Guttmann CR, Barkhof F (2006). Human gray matter: feasibility of single-slab 3D double inversion-recovery high-spatial-resolution MR imaging. Radiology.

[27] Kallmes DF, Hui FK, Mugler JP (2001). Suppression of cerebrospinal fluid and blood flow artifacts in FLAIR MR imaging with a single-slab three-dimensional pulse sequence: initial experience. Radiology.

[28] Chagla GH, Busse RF, Sydnor R, Rowley HA, Turski PA (2008). Three-dimensional fluid attenuated inversion recovery imaging with isotropic resolution and nonselective adiabatic inversion provides improved three-dimensional visualization and cerebrospinal fluid suppression compared to two-dimensional flair at 3 tesla. Investig Radiol.

[29] Kijowski R, Gold GE (2011). Routine 3D magnetic resonance imaging of joints. J Magn Reson Imaging.

[30] Gold GE, Busse RF, Beehler C, Han E, Brau AC, Beatty PJ, Beaulieu CF (2007). Isotropic MRI of the knee with 3D fast spin-echo extended echo-train acquisition (XETA): initial experience. AJR Am J Roentgenol.

[31] Welsch GH, Zak L, Mamisch TC, Paul D, Lauer L, Mauerer A, Marlovits S, Trattnig S (2011). Advanced morphological 3D magnetic resonance observation of cartilage repair tissue (MOCART) scoring using a new isotropic 3D proton-density, turbo spin echo sequence with variable flip angle distribution (PD-SPACE) compared to an isotropic 3D steady-state free precession sequence (true-FISP) and standard 2D sequences. J Magn Reson Imaging.

[32] Piccini D, Littmann A, Nielles-Vallespin S, Zenge MO (2012). Respiratory self-navigation for whole-heart bright-blood coronary MRI: methods for robust isolation and automatic segmentation of the blood pool. Magn Reson Med.

[33] Pang J, Bhat H, Sharif B, Fan Z, Thomson LE, LaBounty T, Friedman JD, Min J, Berman DS, Li D (2014). Whole-heart coronary MRA with 100% respiratory gating efficiency: self-navigated three-dimensional retrospective image-based motion correction (TRIM). Magn Reson Med.

[34] Henningsson M, Smink J, van Ensbergen G, Botnar R (2018). Coronary MR angiography using image-based respiratory motion compensation with inline correction and fixed gating efficiency. Magn Reson Med.

[35] Spuentrup E, Manning WJ, Botnar RM, Kissinger KV, Stuber M (2002). Impact of navigator timing on free-breathing submillimeter 3D coronary magnetic resonance angiography. Magn Reson Med.

[36] Mitsouras D, Mulkern RV, Rybicki FJ (2006). Strategies for inner volume 3D fast spin echo magnetic resonance imaging using nonselective refocusing radio frequency pulses. Med Phys.

[37] Feinberg DA, Hoenninger JC, Crooks LE, Kaufman L, Watts JC, Arakawa M (1985). Inner volume MR imaging: technical concepts and their application. Radiology.

[38] Hussain T, Clough RE, Cecelja M, Makowski M, Peel S, Chowienczyk P, Schaeffter T, Greil G, Botnar R (2011). Zoom imaging for rapid aortic vessel wall imaging and cardiovascular risk assessment. J Magn Reson Imaging.

[39] Hussain T, Lossnitzer D, Bellsham-Revell H, Valverde I, Beerbaum P, Razavi R, Bell AJ, Schaeffter T, Botnar RM, Uribe SA (2012). Three-dimensional dual-phase whole-heart MR imaging: clinical implications for congenital heart disease. Radiology.

[40] Sorensen TS, Korperich H, Greil GF, Eichhorn J, Barth P, Meyer H, Pedersen EM, Beerbaum P (2004). Operator-independent isotropic three-dimensional magnetic resonance imaging for morphology in congenital heart disease: a validation study. Circulation.

[41] Wang J, Yarnykh VL, Hatsukami T, Chu B, Balu N, Yuan C (2007). Improved suppression of plaque-mimicking artifacts in black-blood carotid atherosclerosis imaging using a multislice motion-sensitized driven-equilibrium (MSDE) turbo spin-echo (TSE) sequence. Magn Reson Med.

[42] Zhu C, Graves MJ, Yuan J, Sadat U, Gillard JH, Patterson AJ (2014). Optimization of improved motion-sensitized driven-equilibrium (iMSDE) blood suppression for carotid artery wall imaging. J Cardiovasc Magn Reson.

[43] Viessmann O, Li L, Benjamin P, Jezzard P (2017). T2-weighted intracranial vessel wall imaging at 7 tesla using a DANTE-prepared variable flip angle turbo spin echo readout (DANTE-SPACE). Magn Reson Med.

[44] Stuber M, Botnar RM, Danias PG, Sodickson DK, Kissinger KV, Van Cauteren M, De Becker J, Manning WJ (1999). Double-oblique free-breathing high resolution three-dimensional coronary magnetic resonance angiography. J Am Coll Cardiol.

[45] Nguyen KL, Park EA, Yoshida T, Hu P, Finn JP (2017). Ferumoxytol enhanced black-blood cardiovascular magnetic resonance imaging. J Cardiovasc Magn Reson.

[46] François CJ, Tuite D, Deshpande V, Jerecic R, Weale P, Carr JC (2009). Pulmonary vein imaging with unenhanced three-dimensional balanced steady-state free precession MR angiography: initial clinical evaluation. Radiology.

[47] Krishnam MS, Tomasian A, Malik S, Singhal A, Sassani A, Laub G, Finn JP, Ruehm S (2009). Three-dimensional imaging of pulmonary veins by a novel steady-state free-precession magnetic resonance angiography technique without the use of intravenous contrast agent: initial experience. Investig Radiol.

[48] Liu CY, Bley TA, Wieben O, Brittain JH, Reeder SB (2010). Flow-independent T (2)-prepared inversion recovery black-blood MR imaging. J Magn Reson Imaging.

[49] Ginami G, Neji R, Phinikaridou A, Whitaker J, Botnar RM, Prieto C (2018). Simultaneous bright- and black-blood whole-heart MRI for noncontrast enhanced coronary lumen and thrombus visualization. Magn Reson Med.

