# Supplementary material for: Feasibility of 3D black-blood variable refocusing angle fast spin echo cardiovascular magnetic resonance for visualization of the whole heart and great vessels in congenital heart disease
Source: J Cardiovasc Magn Reson. 2018 Nov 26;20:76. doi: 10.1186/s12968-018-0508-1 (PMC6260764; doi:10.1186/s12968-018-0508-1)
Supplement: Supplementary file 1 — Figure S1. The orientation of the field-of-view determines effectiveness of blood suppression as gradients are only performed in readout direction during startup echoes. As a result, for coronal orientation with readout in foot-head direction, blood signal is well-supressed in the descending aorta while residual signal is present in the aortic arch (arrows). Similarly, for a transverse orientation with readout along the anterior-posterior direction, the blood signal in the aortic arch is better suppressed while significant blood signal can be seen in the descending aorta (arrows). Figure S2. Scan planning of 3D FSE, using a narrower excitation volume than encoding volume in the slice direction (a) to spatially encode potential artifacts arising from the non-selective refocusing pulses. Resulting 3D FSE image shown in slice-encoding direction demonstrating excellent suppression of outer volume signal (b). In-plane images through the inner volume (c) and outer volume (d) shows the effectiveness of outer volume suppression. The signal in the outer volume image is magnified by a factor of 50 to highlight the signal suppression in these vials which ranged in T1 from 250 ms to 1500 ms. (DOCX 495 kb) [file 12968_2018_508_MOESM1_ESM.docx]

**Additional files**


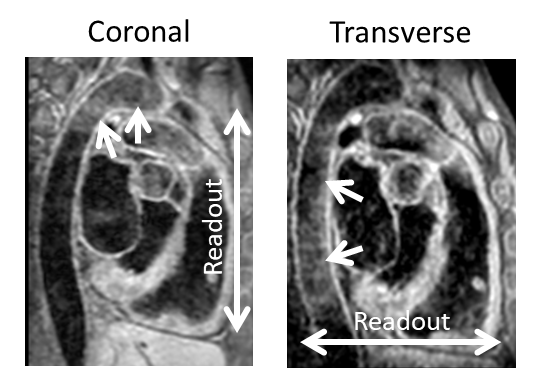


Figure S1. The orientation of the field-of-view determines effectiveness of blood suppression as gradients are only performed in readout direction during startup echoes. As a result, for coronal orientation with readout in foot-head direction, blood signal is well-supressed in the descending aorta while residual signal is present in the aortic arch (arrows). Similarly, for a transverse orientation with readout along the anterior-posterior direction, the blood signal in the aortic arch is better suppressed while significant blood signal can be seen in the descending aorta (arrows).


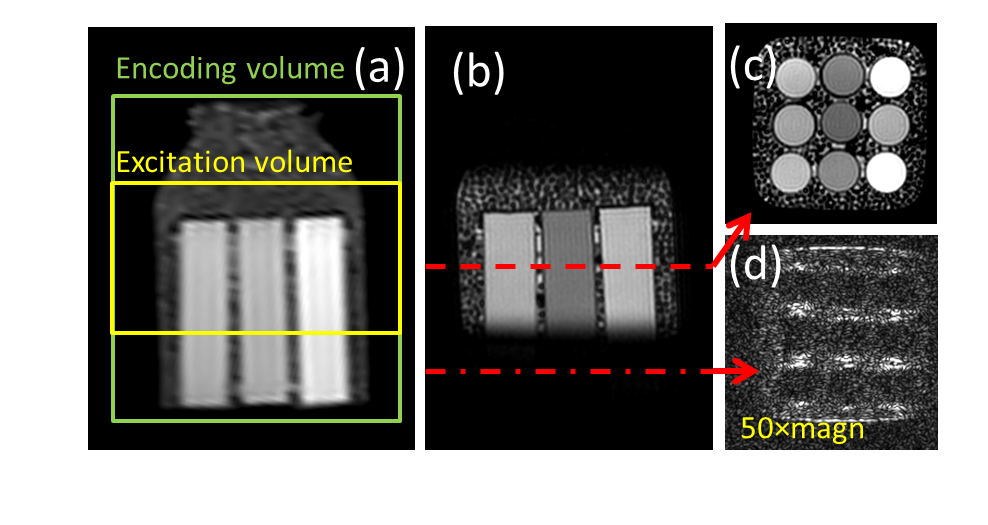


Figure S2. Scan planning of 3D FSE, using a narrower excitation volume than encoding volume in the slice direction (a) to spatially encode potential artifacts arising from the non-selective refocusing pulses. Resulting 3D FSE image shown in slice-encoding direction demonstrating excellent suppression of outer volume signal (b). In-plane images through the inner volume (c) and outer volume (d) shows the effectiveness of outer volume suppression. The signal in the outer volume image is magnified by a factor of 50 to highlight the signal suppression in these vials which ranged in T1 from 250 ms to 1500 ms.
